# Supercritical-CO_2_ extraction, identification and quantification of polyprenol as a bioactive ingredient from Irish trees species

**DOI:** 10.1038/s41598-021-86393-x

**Published:** 2021-04-02

**Authors:** Hadil Alaydi, Peter Downey, Michelle McKeon-Bennett, Tanya Beletskaya

**Affiliations:** 1 Department of Applied Science, Limerick Institute of Technology, Moylish Park, Moylish, Co Limerick Ireland; 2 Athlone Institute of Technology, Dublin Road, Athlone, Co Westmeath Ireland; 3 Shannon Applied Biotechnology Centre, Department of Applied Science, Limerick Institute of Technology, Moylish Park, Moylish, Co Limerick Ireland

**Keywords:** Plant sciences, Analytical biochemistry

## Abstract

This study ascertained the accumulation of polyprenol from four Irish conifer species *Picea sitchensis, Cedrus atlantica ‘Glauca’, Pinus sylvestris* and *Taxus baccata* and one flowering tree *Cotoneaster hybrida* using supercritical fluid extraction with carbon dioxide (SFE-CO_2_) and solvent extraction. The effects of SFE-CO_2_ parameters such as temperature (ranged from 40 to 70 $^\circ{\rm C}$), pressure (ranged from 100 to 350 bars) and dynamic time (from 70 min to 7 h) were analysed on the extraction efficiency of polyprenol. Qualitative and quantitative analysis of polyprenol was examined using high-performance liquid chromatography. Results showed that *P. sylvestris* accumulated the highest polyprenol yield of 14.00  ± $0.4$mg g^−1^ DW when extracted with hexane:acetone (1:1 v/v). However, with SFE-CO_2_ conditions of 200 bars, 70 $^\circ{\rm C}$, 7 h, with absolute ethanol as a cosolvent with a flow rate of 0.05 ml min^−1^, *P. sitchensis* accumulated the highest polyprenol yield of 6.35 ± $0.4$ mg g^−1^DW. This study emphasised the potential application of SFE-CO_2_ in the extraction of polyprenol as an environmentally friendly method to be used in pharmaceutical and food industries.

## Introduction

Plants are a staple of balanced diet and also an important remedial source for various ailments^1,2^. Plants are essential for pharmacological research and drug development, where they are sources of direct therapeutic drugs, precursor materials for drug synthesis, and models for pharmacologically active compounds^3^. One such compound class exploited for the aforementioned reasons are that of polyisoprenoids^4,5^. Polyisoprenoids (polymers of isoprene unit) are linear hydrophobic, branched chains of fatty alcohol^6^. Polyisoprenoids are divided into two main subgroups found in nature differing based on the hydrogenation status of α-terminal double bond in the molecule, polyprenols (α-unsaturated) and dolichols (α-saturated)^4,7,8^ (Supplementary Fig. S1^7^). Polyprenols are long-chain isoprenoid polymers with the formula (C_5_H_8_)_n_OH, that can be found in a wide range of natural sources, including bacteria and plants. In mammals, the mono-saturated dolichols are dominant^4,9,10^. Plants represent the widest range of polyprenols diversity, containing both poly-cis/trans-prenols^6^. Polyprenols are always found as a mixture of homologues differing in the number of isoprene units^11^. The degree of polymerization of polyprenols is specific to plant species, growth stages and cultivation/growth conditions^8^. In the human body polyprenols are metabolised into dolichols and dolichyl phosphate by enzymatic catalysis^11^. In humans, dolichols regulate the permeability and stability of membranes, partaking in the biosynthesis of human glycoproteins^11,12^. Both dolichols and polyprenols are found in the form of esters, carboxylic acids and free alcohol in the cell^13^, while a small portion is found in the form of phosphates^14^.

Polyprenols have non-carcinogenic, non-mutagenic, non-teratogenic and non-toxic effects in humans^15–17^, while also providing significant anti-tumour, anti-anaemia, anti-HIV and anti-hepatitis C effects^18,19^. Polyprenols have also been found to positively affect conditions such as hypertension, high cholesterol, gout, lupus and diabetes, along with other disorders disturbing proper immune functionality^13,20,21^. Moreover, polyprenols serve as a chemotaxonomic marker for systematic families in botanic taxonomy^22^, and also act as a scavenger against oxygen species generated by chemicals or accumulated upon ageing or UV light^23^. Polyprenols extracted from *Ginkgo biloba* leaves showed anti-bacterial effects against *Escherichia coli, Salmonella enterica, Bacillus subtilis*^24^*.* More studies are required on researching plant sources of polyprenol compounds and their derivatives as suggested in a review by Zhang et al. for developing plant extracts into products for therapeutic use^5^. Examples of polyprenol-based products available commercially are ROPREN^25^ and FORTEPREN^26^, both of which extracted from plants. ROPREN consists of a highly purified mixture of polyprenols (patent No. 001521/07 from 12/07/2007) marketed for the treatment of liver disease. Studies suggest that ROPREN has significantly wider applications than liver disease, such as the ability to normalise immune response, normalise cholesterol levels and alleviate the symptoms of Alzheimer’s disease^25,27^. FORTEPREN, polyprenyl phosphate extracted from fir needles belongs to the antiviral drug family with immunomodulating activity and has been suggested as a treatment for herpes disease^26^.

The high cost of extraction and low yield during purification limit the full industrial application of polyprenols. The aim of this study was to screen Irish conifer trees and one flowering tree as a source of polyprenol and utilise SFE-CO_2_ as a green alternative technology to organic solvent extraction. One of the most valuable characteristic of SFE-CO_2_ is the highly reduced, often to zero usage of toxic organic solvents making it ideal for use in pharmaceutical and food-based industries. In this study we optimised SFE-CO_2_ parameters for the maximum polyprenol yield and compared achieved yields against traditional organic solvent extraction. We further aiming to carry out preliminary purification by flash chromatography and profiling of individual isoprene unit by LC–MS.

## Materials and methods

### Chemical and reagents

HPLC grade 2-propanol (> 99.5%), methanol (> 99.9%) and acetonitrile (> 99.9%) and analytical grade ethanol (≥ 99.8%), hexane (≥ 97%), chloroform (> 99.9%), potassium hydroxide, formic acid and acetone (> 99.9%) were purchased from Lennox (Dublin, Ireland). Polyprenol standard mixes range between (C_70_–C_100_ and C_80_–C_125_) were purchased from LGC limited (Teddington, England). Carbon-dioxide (> 99.9) were purchased from BOC (Limerick, Ireland).

### Preparation of plant material

Needle/leave samples were collected from four conifer *Cedrus atlantica ‘Glauca’*, *Picea sitchensis, Pinus sylvestris* and *Taxus baccate* and one flowering tree *Cotoneaster hybrida* from urban areas of county Limerick (Ireland) in October and November, 2016 and 2017. The sample timeframe was selected based on the study carried out by Bajda et al.^28^, which established that the accumulation of polyprenol was the highest at the end of vegetative season. The plant materials were taxonomically authenticated by Professor Trevor Hodkinson in the Botany Department of Trinity Collage Dublin, the University of Dublin. Young and old needles were selected at random from three mature trees of each species and combined. Samples were stored at − 20 $^\circ{\rm C}$ overnight and subsequently freeze-dried (Thermo Scientific Heto Power Dry LL3000, United Kingdom) at − 50 $^\circ{\rm C}$ temperature for two days. When the drying process was completed, samples were ground to a fine powder (~ 0.4–0.8 mm) using a mill (MM 400 Retsch, England) and stored at − 20 $^\circ{\rm C}$ for further analysis.

### Extraction with organic solvent

200 mg of dried, homogenised tissue from each species was extracted three times with 5 ml of 4:1 (v/v) hexane:acetone and 1:1 (v/v) hexane:acetone. After each extraction samples were vortexed and sonicated for 15 min followed by incubation at 40 $^\circ{\rm C}$ for an hour with occasional shaking. The tubes were centrifuged at 3000 rpm for 10 min. The supernatant was collected and placed into new 15 ml plastic tube. The samples were extracted three times, to which 5 ml of fresh solvent was added, incubated at 40 $^\circ{\rm C}$ for an hour and centrifuged at 3000 rpm for 10 min. The supernatants from the three extracts were combined for a total extract volume of approximately 15 ml. All tubes were fully evaporated under a stream of nitrogen and stored at − 20 $^\circ{\rm C}$ for further analysis.

### Supercritical fluid carbon dioxide extraction (SFE-CO_2_)

SFE-CO_2_ was performed on Super Critical Extraction system MV-10 ASCFE which consists of thermocube chiller, fluid delivery module (co-solvent pump and CO_2_ pump), extraction oven (extraction vessels), automatic backpressure regulator (ABPR) and fraction collection module with makeup pump by Waters (Dublin, Ireland). 3 g of powdered sample was placed into a 3 ml sample vessel and extracted under the following parameters: CO_2_ flow rate at 10 ml min^−1^, ethanol co-solvent flow rate at 0.05 ml min^−1^, and make-up solvent flow rate was 0.6 ml min^−1^. All flow rates were kept constant throughout the process. A three factors, four levels orthogonal array design of L_16_ (4^3^)^29^ was employed for the optimisation of extraction parameters, temperature ($^\circ{\rm C}$), pressure (bar), dynamic time (min) (Table 1). Sixteen experiments were performed in order to identify the best parameters for the extraction of polyprenol from selected plant species (Supplementary Table S1).

**Table 1:** The parameters factors and levels of the orthogonal array design L_16_.

Factors	Levels			
	1	2	3	4
Pressure (bar)	100	200	300	350
Temperature ($\boldsymbol{^\circ{\rm C} }$)	40	50	60	70
Dynamic time (min)	40	50	60	70

Further optimisation experiments were carried out to investigate the effect of the extended dynamic time. These extractions were conducted with dynamic time of up to 7 h. The temperature and pressure selections for these further time experiments were 200 bar and 70 $^\circ{\rm C}$. Those temperature and pressure set-points were selected based on the initial development process being the best parameters which resulted in the highest polyprenol yield.

### Saponification of the lipid extracts

All solvent and SFE-CO_2_ extracts were completely dried under nitrogen gas and saponified by adding to each extract 1500 μl of saponification solution (2.5 M KOH in methanol, 1:4 v/v), prior to being vortexed and incubated at 72 $^\circ{\rm C}$ for 15 min. Following incubation, 225 μl of formic acid was added, 1725 μl of chloroform and 375 μl of milli-Q water. The final mixture was vortexed and centrifuged for 5 min at 3000 rpm where two layers formed. The organic layer containing free fatty acid and polyprenol was transferred to a clean vial, dried and stored at − 20 $^\circ{\rm C}$ for further analysis ^30^.

### Sample/standard preparation for HPLC

Solvent and SFE-CO_2_ extracts were evaporated to dryness under a stream of nitrogen and reconstitute in 5 ml hexane and filtered through 0.20 μm filter before HPLC analysis. Two sets of polyprenol standard mixtures, quantitative mixture of C70–C100 prenologues (P-14 to P-20) and quantitative mixture of C80–C125 prenologues (P-16 to P-25) were used for the HPLC analysis, provided by LGC in liquid form at a concentration of 5 mg/0.5 ml hexane, serial dilutions were prepared.

### High performance liquid chromatography (HPLC)

Polyprenol identification and quantification was preformed via HPLC according to a protocol previously described by Yu et al.^31^ with some modifications. Analysis was performed on an Agilent Technologies HPLC consisting of G1329B auto-sampler, G1316B thermostatted column compartment, G1315C DAD detector, G1312B binary pump and G1379B degasser. Separation was carried out on a ZORBAX Eclipse Plus C18 4.6 × 150 mm, 5 μm column. The mobile phase consisted of 100% acetonitrile (solvent A) and 100% isopropanol (solvent B) using the following gradient: 0–5 min—40%B; 6–35 min—40–75% B; 36–50 min—40% B; flow rate of 0.5 ml min^−1^; column temperature of 40 $^\circ{\rm C}$; detection via UV-DAD at 210 nm, and an injection volume of 10 µl ^31^.

### Statistical analysis

Each experiment was carried out in triplicate and the data were expressed as means ± standard deviation (SD). Statistical analysis were prerformed on Microsoft Excel (Version 16.39, Microsoft 365 subscription, data analysis 2019 software). The analysis of variance (ANOVA) with a p-values less than 0.05 were considered to be statistically significant. Regression analysis (t-test) was performed to test the significance of the independent variables and one way ANOVA using STATA (Stata Statistical Software, version 13.0, Collage Station, TX: StataCorp LLC).

## Results and discussion

### Identification and quantification of polyprenol

Across many cited studies, organic solvents have been investigated for their effects on both the yield and purity of polyprenols extracts including ethyl ether, hexane/acetone (4:1 v/v, 1:1 v/v, and 1:9v/v), and ethyl acetate^32,33^. Another study investigating the extraction of polyprenols from *Ginkgo biloba* leaves showed that petroleum ether gave the cleanest extract, with a yield of 0.92 g DW and purity of 18.3%, while hexane/acetone mix (4:1, v/v) gave the highest yield of 1.16 g DW and a purity of 18.3%^32^. Therefore, in the current study needles from *C. atlantica ‘Glauca’, P. sitchensis, P. sylvestris* and *T. Baccata* and leaves from *C. hybrida* were extracted using SFE-CO_2_ and a mixture of hexane:acetone at two ratios of (4:1; 1:1 v/v).

The identification of polyprenol compounds from the needles and leaves of conifers/flowering species were based on UV spectral data (210 nm) with the aid of external standards. Polyprenol was naturally present as a mixture of homologues which make it difficult to separate and quantify. Solvent extracts with hexane:acetone (4:1; 1:1 v/v) and 16 different combination of SFE-CO_2_ parameters (Supplementary Table S1) were optimised for *C. atlantica ‘Glauca’.* For the purpose of optimisation of the extraction method, only one plant material was chosen at random i.e. *C. atlantica ‘Glauca’*. Selection was based on availability and consistency within the extraction techniques.

**Figure 1:**
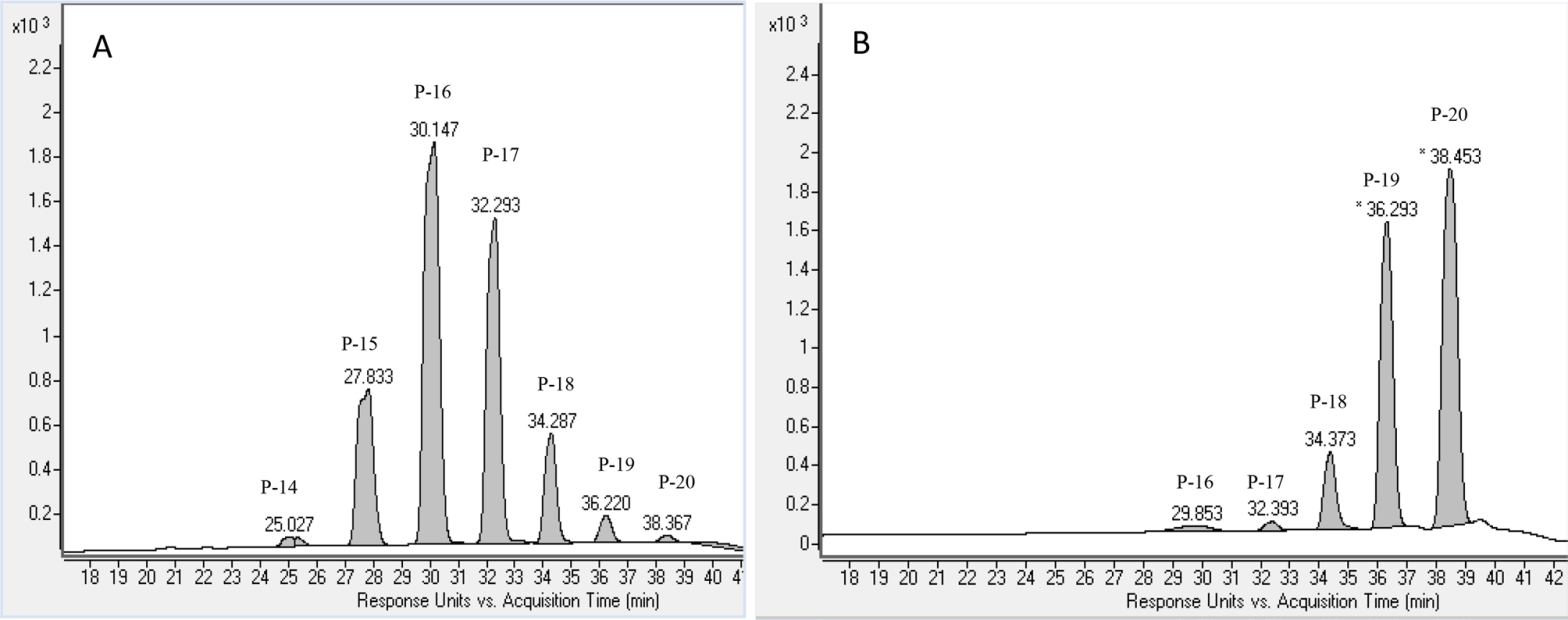
HPLC chromatogram of polyprenol standard mixture of (**A**) C_70_–C_100_ prenologues (P14–P20) and (**B**) C_80_–C_100_ prenologues (P16–P20). Where ‘P’ prenologues (number of isoprene unit present), ‘C’ is the number of carbons in polyprenol chain. UV at 210 nm [PX5 = carbon chain length].

The polyprenol content present in the samples were compared against two sets of standard mixtures, quantitative mixture of C70–C100 prenologues (P-14 to P-20) and quantitative mixture of C80–C125 prenologues (P-16 to P-25) (Fig. 1). Both sets of standards overlapped with regards to the length of the polyprenols tested ensuring adequate identification with a wide range of lengths. As mentioned previously polyprenol from plant material has different cis/trans configuration. These configurations affect the retention time and make it difficult to confirm the exact identity of these polyprenol. However, these variations were small in comparison to the chain length and stereochemistry can affect chromatographic separation.

### Evaluation of extraction procedures for polyprenol content of *Cedrus atlantica ‘Glauca’*

**Figure 2:**
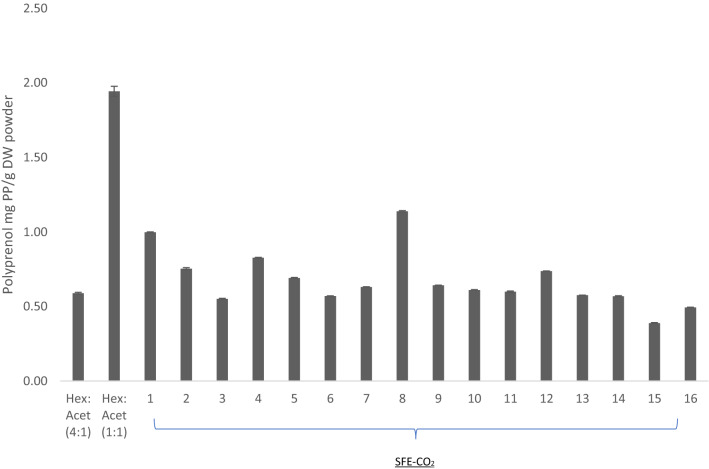
Quantitive analysis of the total polyprenol content (mg g^−1^DW) extracted from C. atlantica ‘Glauca’ under two extraction methods; solvent using hexane:acetone (4:1; 1:1, v/v) and 16 different combination of SFE-CO_2_ parameters without saponification. The polyprenol content from SFE-CO_2_ extractions were based on a dynamic time of 70 min and also without applying saponification on both extraction methods. Each value presents the mean value ± SD. Figure was prepared using Microsoft excel (Version 16.39, Microsoft 365 subscription, data analysis 2019 software).

Two solvent extracts and extracts obtained from 16 different SFE-CO_2_ parameter permutations of *C. atlantica ‘Glauca’* were analysed by HPLC for qualitative and quantitative determination of polyprenol. HPLC analysis allowed for the determination of total polyprenol content as well as the range of isoprene units according to chain length. The results of a qualitative evaluation of isoprene chain length in the needles of *C. atlantica ‘Glauca’* was shown in Table 2. The quantitative determination of the total polyprenol content in mg g^−1^dry weight was shown in Fig. 2. Variation in polyprenol chain length was evident between extraction types, indicating differences in selectivity between the methods. These differences were observed in *C. atlantica ‘Glauca’* where it accumulated polyprenol with medium to high chain length from C95 extracted by hexane:acetone (4:1) and C75–C100 extracted by hexane:acetone (1:1).

**Table 2:** Polyprenol chain length identified from HPLC chromatogram in *C. atlantica* ‘Glauca’*.*

Solvent extraction	Prenologues (number of isoprene units present)						
hexane:acetone (4:1 v/v)						P-19	P-20
hexane:acetone (1:1 v/v)		P-15		P-17	P-18	P-19	P-20

SFE-CO_2_ parameters (*T* °C, *P* bar, *t* mins)	Prenologues (number of isoprene units present)						
1 (40, 100, 40)	P-14	P-15	P-16		P-18	P-19	P-20
2 (50, 100, 50)		P-15	P-16		P-18	P-19	P-20
3 (60, 100, 60)		P-15	P-16		P-18	P-19	P-20
4 (70, 100, 70)	P-14		P-16	P-17	P-18	P-19	P-20
5 (40, 200, 40)				P-17	P-18	P-19	P-20
6 (50, 200, 50)					P-18	P-19	P-20
7 (60, 200, 60)	P-14				P-18	P-19	P-20
8 (70, 200, 70)	P-14	P-15	P-16		P-18	P-19	P-20
9 (40, 300, 40)	P-14				P-18	P-19	P-20
10 (50, 300, 50)	P-14				P-18	P-19	P-20
11 (60, 300, 60)					P-18	P-19	P-20
12 (70, 300, 70)	P-14	P-15			P-18	P-19	P-20
13 (40, 350, 40)	P-14				P-18	P-19	P-20
14 (50, 350, 50)	P-14		P-16		P-18	P-19	P-20
15 (60, 350, 60)	P-14				P-18	P-19	P-20
16 (70, 350, 70)	P-14				P-18	P-19	P-20

The plant needles were extracted with two different extraction solvent and under 16 different SFE-CO_2_ parameter combinations.

The polyprenol chain length identified in SFE-CO_2_ of *C. atlantica ‘Glauca’* extracts ranged from C70 to C100 (P-14 to P-20). Treatment number 8, with conditions of 200 bar, 70 $^\circ{\rm C}$, 70 min and ethanol modifier (0.05 ml min^−1^), were identified as the best parameters at extracting the longer chains of polyprenol. A wide range of isoprene unit lengths were detectable across the SFE-CO_2_ conditions investigated with lengths of P-20 dominating, followed by P-19 and P-18 isoprene unit.

**Figure 3:**
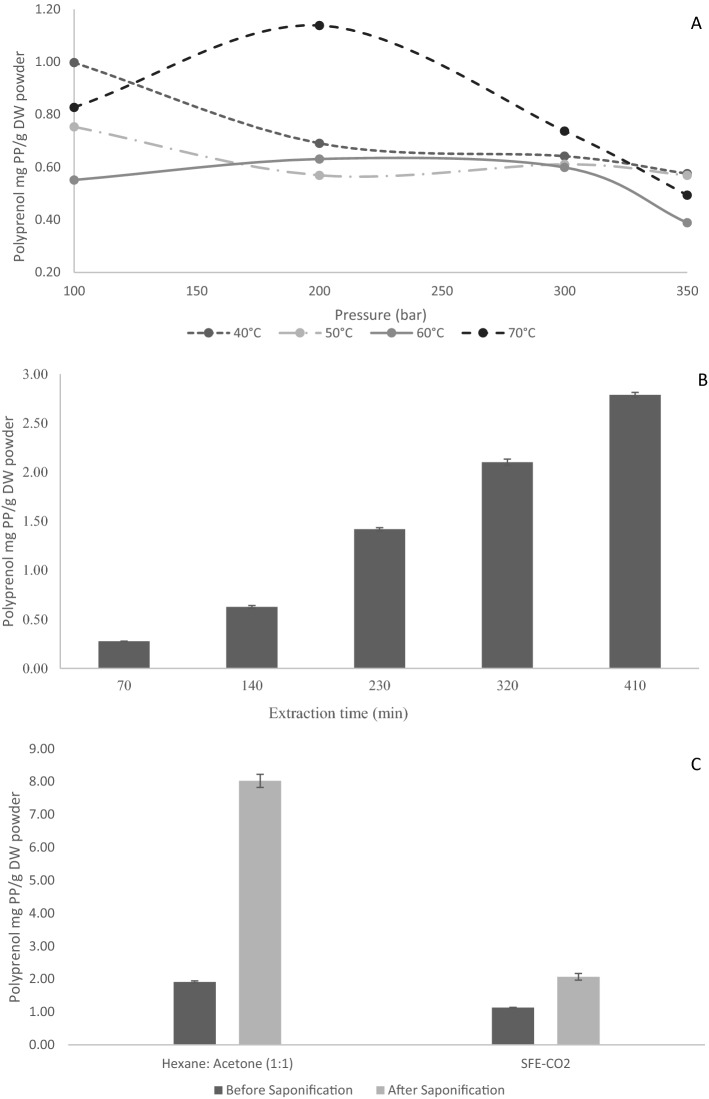
The effect of SFE-CO_2_ parameters (**A**) effect of pressure and temperature and (**B**) dynamic time (**C**) effect of saponification on the total polyprenol content in mg/g DW at a constant temperature of 70 $^\circ{\rm C}$ and pressure of 200 bar with a presence of ethanol as co-solvent. During the time course of the SFE-CO_2_ of *C. atlantica ‘Glauca’* extracts were collected at different time intervals and samples were analysed for total polyprenol using HPLC. Each value presents the mean value ± SD. Figures prepared using Microsoft excel (Version 16.39, Microsoft 365 subscription, data analysis 2019 software).

The identification and quantification of the total polyprenol content concluded that hexane:acetone (1:1; v/v) and SFE-CO_2_ treatment 8 had the highest polyprenol content of 1.94 $\pm$ 0.03 mg g^−1^DW and 1.14 ± 0.004 mg g^−1^DW, respectively. The effect of pressure (bar) and temperature ($^\circ{\rm C}$) were analysed see Fig. 3A. An increase in polyprenol content was observed (p < 0.05) from 0.28 ± 0.001 to 2.78 ± 0.02 mg g^−1^DW as the dynamic time was increased from 70 min to 7 h, as depicted in Fig. 3B. These results correlate with data presented by Jozwiak et al.^13^; as extraction time increases so does the content of polyprenol. During their extraction of *Sorbus* spp., the amount of polyprenol increased slowly, in proportion to time and was observed to require at least 56 h to achieve the maximum yield. Extraction times were much quicker from *Spruce* spp., for which maximum yield was achieved in 7 h^13^. The effect of time would also depend on the compositional nature of the needles/leaves of the species selected. Wang et al. showed that extraction time inversely affects the extract composition; compounds of low molecular mass and which were less polar tend to be extracted faster, since the extraction mechanism was controlled by internal diffusion^34^.

In the SFE process, CO_2_ is small and linear in structure molecule which increases its permeability yet, one of its limitation include non-polar and its capacity to form specific interaction between solvent and solute. Adding polar co-solvent improves the CO_2_ polarity; this will enhance the solubility power and extract more polyprenol content. In this study ethanol as a co-solvent at a flow rate of 0.05 ml/min and kept constant however the volume of ethanol varies across different extraction time, for example at 40 min a total volume of 2 ml ethanol was used in comparison to 3 ml at 70 min extraction time. Other types of co-solvent with higher polarity than ethanol can be applied such as acetone. However, the aim was to use an eco-friendly process for the extraction of polyprenol hence applying SFE and moreover ethanol as a modifier. Ethanol is less toxic and of lower risk to human health and normally accepted in pharmaceutical as per FDA regulation. In addition, avoiding the use of toxic chemicals as a modifier (such as acetone), eliminates the purification step after SFE extraction.

The optimisation of the SFE-CO_2_ process depends on multiple parameters which affect the solubility of polyprenol in supercritical fluid (SF). Based on the literature, there are data available regarding the solubility of solid-supercritical fluid equilibrium system. However, no solubility data has been reported on polyprenol-supercritical carbon dioxide system. There are different models to study and estimate the solubility of solute-supercritical fluid including, solubility parameter model, semiempirical methods, equation of state and molecular dynamic simulation^35^. In the current study the solubility of polyprenol in carbon dioxide was correlated with other parameters such as pressure, temperature, CO_2_ density and co-solvent. Other factors, which affect the solubility are the solute molar mass, polarity and vapour pressures. At a constant temperature, an increase in pressure enhances the solvating power; and at a constant pressure, an increase in the temperature reduces the solvating power and the solvent density. High solvent flow rate increases the capacity of extraction of solute from plant, however in some cases it causes a decrease in the extraction yield, due to less time allowed for solvent–solute interaction^36^.

Supercritical fluids possess thermal and chemical properties between those of a pure gas and a liquid which makes them capable of dissolving other materials. CO_2_ has a low viscosity as a gas, high density as liquid and intermediate level of diffusion between those of gases and liquids at which densities are similar. Addition of a highly polar cosolvent, will increase its solvating power and improve its polarity. In this study, ethanol is used as a cosolvent as it is less toxic and of low risk to human health and mostly used in pharmaceutical as per FDA.

Solvent density plays a critical role on the solute solubility in supercritical fluid due to its solvating power at critical conditions rather than solute-vapour pressure hence, density decreases with an increase in temperature as seen in Supplementary Table S2. At a higher pressure, CO_2_ density increases which increases solubility and the intermolecular distances between CO_2_ molecules reduced, this will increase solute–solvent interaction. Based on the crossover behaviour of solubility isotherms, at a pressure below the lower crossover pressure and above the upper crossover pressure, the solubility increases with increase in temperature hence higher solid-vapour pressure. At pressure between lower and upper crossover pressure, solubility increases as a result of rapid decrease in solvent density; known as ‘retrograde vaporisation’. At the lower and upper crossover pressure the effect of solute vapour pressure and solvent density on solid solubility is equal. The crossover pressure is at the point where the slope of the solubility versus temperature change^37^.

In Fig. 3A, at the pressures of 100, 300 and 350 bars across the temperature studied, with exception to 70 $^\circ{\rm C}$, as the pressure range between lower crossover pressure and upper crossover pressure. Where, as temperature increase, solubility of solute in solvent decrease hence lower content of polyprenol. This is a result of interaction effect of CO_2_ density and solute vapour pressure. However an exception to the above phenomenon observed at 200 bar where the solubility of solute in solvent increase as the temperature increased to 70 $^\circ{\rm C}$. To account for this anomaly, at a pressure above crossover pressure, the solute vapour pressure effect became dominant and solubility increase when temperature increase.

In this study, the effect of saponification on the quantification of the total polyprenol content was also analysed, as indicated in Fig. 3C. Saponification in this case is a pre-treatment of the samples to release the compound of interest or remove some interfering components and, therefore, enhance the HPLC analysis of polyprenol. A significant increase (p < 0.05) in polyprenol content was observed with approximately 21% increase in both extraction techniques. Regression analysis was performed to test the significance of the independent variables using t-test and p-values (See Supplementary Tables S3 and S4). According to the p-values the variables pressure (bar) and standard deviation are significant according to the 95% confidence intervals where p < 0.05.

### Screening other conifer and flowering trees for the accumulation of polyprenol

Based on the identification of polyprenol from both solvent and SFE-CO_2_ extraction, the best solvent and optimum SFE-CO_2_ conditions were defined to be hexane:acetone (1:1 v/v), and 200 bars, 70 $^\circ{\rm C}$, 7 h and 100% ethanol (0.5 ml min ^−1^). The above extractions conditions were further applied to the needles of *T*. *bacatta*, *P. sylvestris*, and *P. sitchensis* and leaves of *C. hybrida.*

The results of qualitative analysis of polyprenol chain length identified in the needles/leaves of the species studied were shown in Table 3. Among all plants, the polyprenols composed of P-14 to P-20 of isoprene units were detectable with P-19 dominating followed by P-20. The extraction techniques effect the isoprene identified within the same plant species. For example *T. baccata* extracted with hexane:acetone (1:1, v/v) comprised more isoprene unit in comparison to SFE-CO_2_ which only comprised P-19. All HPLC chromatograms of the plants species extracted by both techniques and the composition isoprene chain length can be detected are presented in Table 4.

**Table 3:**
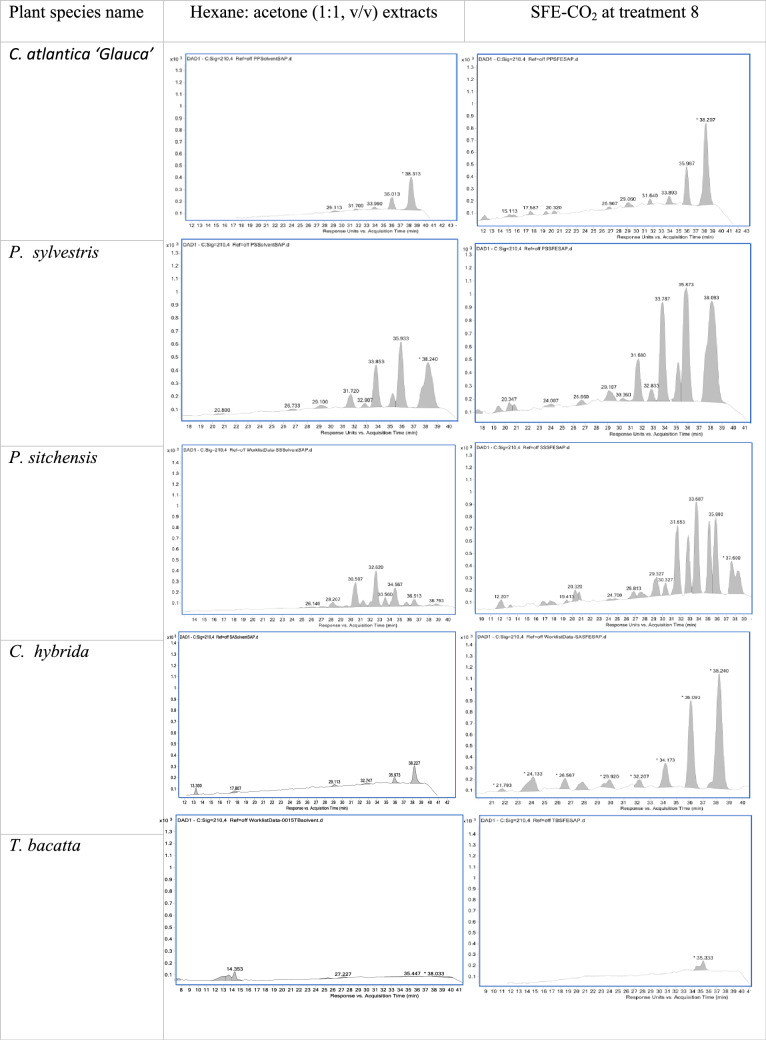
Chromatograms of plant tissue extracted with hexane:acetone (1:1, v/v) solvent mix and SFE-CO_2_ at 200 bar, 70 $\mathrm{^\circ{\rm C} }$, 7 h and 100% ethanol as a modifier.

**Table 4:** Polyprenol chain length identified from HPLC chromatogram in *P. sitchensis, P. sylvestris, C. hybrida* and *T. baccata*.

Hexane:acetone (1:1 v/v)	Prenologues (number of isoprene units present)									
*P. sitchensis*		P-15	P-16	P-17	P-18		P-19		P-20	
*P. sylvestris*	P-14	P-15	P-16	P-17	P-18		P-19		P-20	
*C. hybrida*				P-17	P-18		P-19		P-20	
*T. baccata*	P-14	P-15	P-16						P-20	

SFE-CO_2_ (treatment 8)	Prenologues (number of isoprene units present)									
*P. sitchensis*	P-14	P-15	P-16	P-17	P-18		P-19		P-20	
*P. sylvestris*	P-14	P-15	P-16	P-17	P-18		P-19		P-20	
*C. hybrida*	P-14	P-15	P-16	P-17	P-18		P-19		P-20	
*T. baccata*							P-19			

The plant needles/leaves were extracted with hexane:acetone (1:1 v/v) and SFE-CO_2_ treatment 8 (200 bar, 70 $\mathrm{^\circ{\rm C} }$, 7 h and 100% ethanol as a modifier).

**Figure 4:**
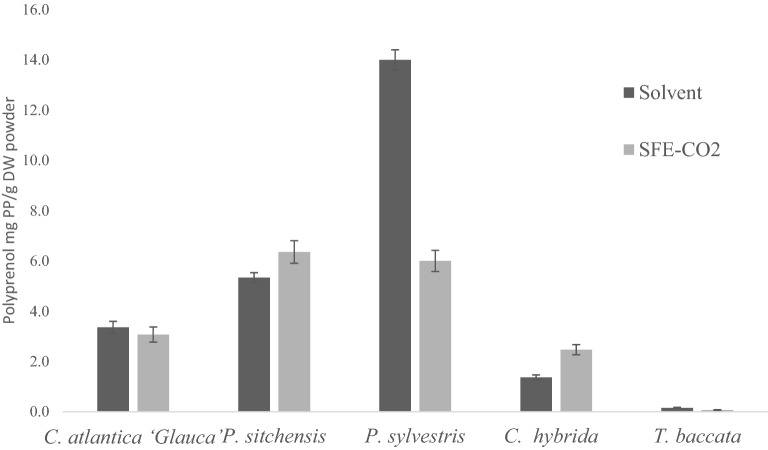
The total polyprenol content extracted by both extraction method (mg/g DW) calculated for *C. atlantica ‘Glauca’, P. sitchensis, P. sylvestris, C. hybrida* and *T. baccata* Each value presents the mean value ± SD. Figure prepared using Microsoft excel (Version 16.39, Microsoft 365 subscription, data analysis 2019 software).

Quantification of the total polyprenol content in mg g^−1^ DW was calculated based on the total peak areas of all polyprenol isoprene chain length identified. SFE-CO_2_ resulted in the extraction of a wider variety of isoprene chain lengths when compared to solvent extraction, but resulted in lower polyprenol yields in some of the plant species studied as depicted in Fig. 4. The total polyprenol content in the needles/leaves of plants studied was shown to be as high as 1.2% of the dry weight.

Polyprenol content in the total SFE-CO_2_ extracts were 3.07 ± 0.3 mg g^−1^DW, 6.35 ± 0.4 mg g^−1^DW, 6.00 ± 0.4 mg g^−1^DW, 2.47 ± 0.2 mg g^−1^DW and 0.06 ± 0.01 mg g^−1^DW for *C. atlantica ‘Glauca’*, *P. sitchensis*, *P. sylvestris*, *C. hybrida* and *T. baccata* respectively. For the organic solvent, polyprenol content in the respective extracts were 3.35 ± 0.24 mg g^−1^DW, 5.34 ± 0.2 mg g^−1^DW, 14.00 $\hspace{0.17em}\pm \hspace{0.17em}0.4$ mg g^−1^DW, 1.37 ± 0.1 mg g^−1^DW and 0.16 ± 0.02 mg g^−1^DW, respectively.

### General discussion

Based on the results of the current study, the plant species screened proved to be rich sources of polyprenols. The polyprenols in species such as *P. sylvestris*^38,39^ and *T. baccata*^40^ have been characterised previously; however, to the best of the author’s knowledge, this was the first record of *P. sitchensis, C. hybrida* and *C. atlantica ‘Glauca’* screened for the accumulation of polyprenols. This is also the first record investigating the use of SFE-CO_2_ as an extraction technique for these polyprenols from the listed plant species.

In the current study, the highest yield obtained was 14.00 ± 0.4 mg g^−1^ DW from *P. sylvestris* using hexane:acetone (1:1, v/v). *P. sylvestris* accumulated polyprenol with medium to high chain lengths ranging from C_70_–C_100_. In previous literature, petroleum ether was used to extract polyprenol from the old needles of *Taxus chinensis var. mairei*, resulting in 30 mg g^−1^DW yield^31^. Ethyl acetate was used to optimize the extraction yield of polyprenol from *Cunninghamia lanceolate* needles in terms of extraction time, temperature, and liquid—solid ratio. The percentage yield was 1.22 ± 0.04% under the following condition (71.4 $^\circ{\rm C}$, 5.96 h and 9.3 mg ml^-1^)^33^. Hexane:acetone (1:1, v/v) was used to extract *Sorbus intermedia*, *Picea abies* and *Nicotiana tabacum* and resulted in 6.7 ± 0.5, 10 ± 0.6 and 2.1 ± 0.2 mg g^−1^DW^13^. Various species from the *Lauraceae* family were subjected to hexane:acetone (1:1, v/v) for the extraction of polyprenol with resulting yields ranging from 0.06 to 3%^41^.

Research presented by Jozwiak et al.^13^ illustrated optimum conditions were 210 bar and 60 $^\circ{\rm C}$ giving a CO_2_ density of 736.4 kg/m^3^, whereas the optimum conditions of current study were 200 bar and 70 $^\circ{\rm C}$ giving a CO_2_ density of 736.7 kg/m^3^. However, due to the differences in the extraction time applied and the plant species screened by both studies, direct comparison was not applicable since these differences effect the accumulation of polyprenol.

## Conclusion

In this study, the extraction, identification and quantification of polyprenol as a health promoting bioactive ingredient from plant needles/leaves was investigated. Two solvent extraction methods (hexane:acetone 4:1; 1:1 v/v) and 16 different parameters of SFE-CO_2_ were applied to *C. atlantica ‘Glauca’ and* the optimal conditions were identified. SFE-CO_2_ extraction was three time less efficient based on polyprenol total yield content, however gave a wider range of isoprene chain lengths. *P. sylvestris* was identified to be the best source of polyprenol among the plant species studied showing a resultant yield of 14.00  ± $0.4$ mg g^−1^DW by hexane:acetone 1:1 v/v. However, the highest resultant yield of 6.35 ± 0.4 mg g^−1^DW by SFE-CO_2_ was obtained from *P. sitchensis.* Due to the wide applications for polyprenol from natural sources, environmentally friendly extraction technique such as SFE-CO_2_ will be an appropriate method for the food, pharmaceutical and nutraceuticals industries.

## Supplementary Information

Supplementary Information.
