# Supplementary material for: Supercritical-CO2 extraction, identification and quantification of polyprenol as a bioactive ingredient from Irish trees species
Source: Sci Rep. 2021 Apr 2;11:7461. doi: 10.1038/s41598-021-86393-x (PMC8018978; doi:10.1038/s41598-021-86393-x)
Supplement: Supplementary file 1 — Supplementary Information. [file 41598_2021_86393_MOESM1_ESM.docx]

**Supplementary Information**

**Supercritical-CO_2_ extraction, identification and quantification of polyprenol as a bioactive ingredient from Irish trees species**

**Hadil Alaydi*^1^, Peter Downey^1^, Michelle McKeon-Bennett^2^, Tanya Beletskaya^1^**

^1^ *Department of Applied Science, Limerick Institute of Technology, Moylish Park, Moylish, Co Limerick, Ireland*

^2^ *Athlone Institute of Technology, Dublin Road, Athlone, Co Westmeath, Ireland.*

Hadil Alaydi^1^ (E-mail: [Hadil.alaydi@lit.ie](mailto:Hadil.alaydi@lit.ie)). Peter Downey^1^ (E-mail: [Peter.Downey@lit.ie](mailto:Peter.Downey@lit.ie)) Michelle McKeon-Bennett^2^ (E-mail: [mmckeonbennett@ait.ie](mailto:mmckeonbennett@ait.ie)) Tanya Beletskaya^1^ (E-mail: [Tanya.Beletskya@lit.ie](mailto:Tanya.Beletskya@lit.ie))

***Corresponding Author (Postal address)**

^1^ Hadil Alaydi

Shannon Applied Biotechnology Centre, Department of Applied Science, Limerick Institute of Technology, Moylish Park, Moylish, Limerick, Ireland.

Tel: +353838533085. E-mail: [hadil.alaydi@lit.ie](mailto:hadil.alaydi@lit.ie)

^2^ Tanya Beletskaya

Department of Applied Science, Limerick Institute of Technology, Moylish Park, Moylish, Limerick, Ireland.

Tel: +35383879056375. E-mail: [tanya.beletskya@lit.ie](mailto:tanya.beletskya@lit.ie)

**Figure Captions and Figures**

**Figure S1**: Structures of polyprenols and dolichols.

**Tables Captions and Tables**

**Table S1**: Supercritical fluid extraction parameters optimised include, pressure (bar), temperature ($℃$) and dynamic time (minutes) along with the total polyprenol content obtained in ($mg$/ g DW)

Table S2: Represent carbon dioxide (CO_2_) density at different combination of temperature and pressure.

**Table S3**: This table gives a correlation coefficient of all factors involved in the optimisation of SFE process. As well as regression analysis performed to test the significance of the independent variables.

**Table S4**: Analysis of variance (ANOVA) independent variables for the extraction of polyprenol from *C. atlantica ‘Glauca’.*


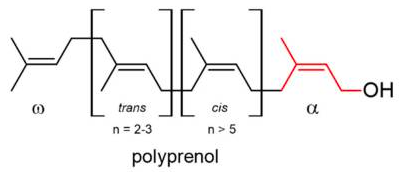

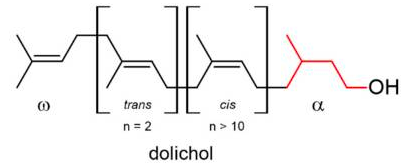


**Figure S1**: Structures of polyprenols and dolichols. Where c and t are the number of cis and trans isoprene units, and α and ω represent the terminal isoprenoid residues. Polyprenols contain all trans or both cis and trans (top). Dolichols contain single bond on α isoprene unit. Both polyprenols and dolichols contain a hydrophilic part at the α-position and hydrophobic at the ω-position ( Adapted from Surowiecki et al. 2019).

**Table S1:** Supercritical fluid extraction parameters optimised include, pressure (bar), temperature ($℃$) and dynamic time (minutes) along with the total polyprenol content obtained in ($mg$/ g DW)

| Treatment | Pressure (bar) | Temperature ($\boldsymbol{℃}$) | Dynamic Time (min) | Polyprenol content $\boldsymbol{m}$g/g DW $\boldsymbol{\pm}$ SD |
| --- | --- | --- | --- | --- |
| 1 | 100 | 40 | 40 | 0.998$\pm$ 0.002 |
| 2 | 100 | 50 | 50 | 0.754 $\pm$ 0.007 |
| 3 | 100 | 60 | 60 | 0.551$\pm$ 0.003 |
| 4 | 100 | 70 | 70 | 0.827$\pm$ 0.002 |
| 5 | 200 | 40 | 40 | 0.691$\pm$ 0.004 |
| 6 | 200 | 50 | 50 | 0.569$\pm$ 0.002 |
| 7 | 200 | 60 | 60 | 0.631$\pm$ 0.001 |
| 8 | 200 | 70 | 70 | 1.139$\pm$0.004 |
| 9 | 300 | 40 | 40 | 0.642$\pm$0.001 |
| 10 | 300 | 50 | 50 | 0.611 $\pm$0.002 |
| 11 | 300 | 60 | 60 | 0.599$\pm$0.004 |
| 12 | 300 | 70 | 70 | 0.737$\pm$0.001 |
| 13 | 350 | 40 | 40 | $0.576\pm$0.004 |
| 14 | 350 | 50 | 50 | 0.569$\pm$0.003 |
| 15 | 350 | 60 | 60 | 0.389$\pm$0.003 |
| 16 | 350 | 70 | 70 | 0.494$\pm$0.001 |

**Table S2:** Represent carbon dioxide (CO_2_) density at different combination of temperature and pressure.

| Treatment | Pressure (bar) | Temperature ($\boldsymbol{℃}$) | Carbon dioxide density (kg/m^3^) |
| --- | --- | --- | --- |
| 1 | 100 | 40 | 628.7 |
| 2 | 100 | 50 | 384.4 |
| 3 | 100 | 60 | 290.0 |
| 4 | 100 | 70 | 255.8 |
| 5 | 200 | 40 | 839.9 |
| 6 | 200 | 50 | 784.4 |
| 7 | 200 | 60 | 723.8 |
| 8 | 200 | 70 | 658.9 |
| 9 | 300 | 40 | 910.0 |
| 10 | 300 | 50 | 870.6 |
| 11 | 300 | 60 | 830.0 |
| 12 | 300 | 70 | 788.0 |
| 13 | 350 | 40 | 934.9 |
| 14 | 350 | 50 | 899.4 |
| 15 | 350 | 60 | 863.2 |
| 16 | 350 | 70 | 826.3 |

**Table S3:** This table gives a correlation coefficient of all factors involved in the optimisation of SFE process. As well as regression analysis performed to test the significance of the independent variables. Figured prepared using STAT (Stata Statistical Software, version 13.0, Collage Station, TX: StataCorp LLC; <https://www.stata.com/>)

**Table S4:** Analysis of variance (ANOVA) independent variables for the extraction of polyprenol from *C. atlantica ‘Glauca’.* Figure prepared using STAT (Stata Statistical Software, version 13.0, Collage Station, TX: StataCorp LLC; <https://www.stata.com/>)
